# Semi-Blind Error Resilient SLM for PAPR Reduction in OFDM Using Spread Spectrum Codes

**DOI:** 10.1371/journal.pone.0127639

**Published:** 2015-05-27

**Authors:** Amr M. Elhelw, Ehab F. Badran

**Affiliations:** Department of Electronics and Communication Engineering, College of Engineering and Technology, Arab Academy and Science and Technology and Maritime Transport, Alexandria 21937, Egypt; Nankai University, CHINA

## Abstract

High peak to average power ratio (PAPR) is one of the major problems of OFDM systems. Selected mapping (SLM) is a promising choice that can elegantly tackle this problem. Nevertheless, side information (SI) index is required to be transmitted which reduces the overall throughput. This paper proposes a semi-blind error resilient SLM system that utilizes spread spectrum codes for embedding the SI index in the transmitted symbols. The codes are embedded in an innovative manner which does not increase the average energy per symbol. The use of such codes allows the correction of probable errors in the SI index detection. A new receiver, which does not require perfect channel state information (CSI) for the detection of the SI index and has relatively low computational complexity, is proposed. Simulations results show that the proposed system performs well both in terms SI index detection error and bit error rate.

## Introduction

Orthogonal Frequency Division Multiplexing (OFDM) is a spectrally efficient multi-carrier modulation technique which is renowned for its ability to elegantly handle selective fading. However, a major drawback of OFDM systems is the high peak-to-average power ratio (PAPR). Several techniques have been proposed to tackle the high PAPR problem ([1–9] and the references therein). Selected mapping (SLM) [4] is one of the efficient techniques which can reduce the PAPR. In SLM techniques, the original data block is converted into several independent signals. The signal with the lowest PAPR is transmitted. However, the selected signal SI index has to be transmitted to enable data recovery at the receiver side, thereby reducing overall throughput.

Recently, various SLM techniques that do not explicit sent the SI index have been published. In [4] and [5], pilot-aided SLM system is proposed where the SI index is embedded in the pilot symbols that are adopted channel estimation. The SI index is detected by examining the autocorrelation between adjacent pilot sub-channel responses [4]. The computational complexity of such a system is relatively high and the effect of modifying the pilot symbols on the channel estimation process has not been investigated. A SLM technique that embedded the SI index in the transmitted data symbol using codes is proposed in [7,8] and [9]. These techniques have drawbacks including that the utilized codes is generated using permutations with no coding properties, increasing energy per transmitted symbol and computational complexity at the receiver side. All systems introduced in [4,7,8] and [9] require prefect CSI for the detection of the SI index. In this paper, spread spectrum codes such as Pseudo-Random Noise PN sequences are embedded in the transmitted symbols. Spread Spectrum codes are known for its sensitivity and selectivity [10] which results in an error resilient SLM technique that can correct possible errors in the recovery of the SI index. The proposed transmitter uses no extra energy for embedding the codes in the transmitted symbol. A novel receiver has also been proposed that includes an auto-correlator receiver. The rest of this paper is organized as follow: Section II gives a detailed exploration of the proposed semi-blind SLM system. Analysis of the SI detection error rate for the proposed SLM is presented in section III. System analysis and simulation results are then given in section IV. Finally, conclusions are drawn in section V.

## The proposed Semi-blind SLM System

Consider an OFDM system utilizes *N* orthogonal subcarriers. In classical SLM, an OFDM data block $X\;=\;{\left\{x_{n}\right\}}_{n\;=\;0}^{N-1}$, consists of *N* complex symbols, is processed to produce *U* possible sequences ${\left\{X_{u}\right\}}_{u\;=\;0}^{U-1}$ as follow

$$X_{u}\;=\;B_{u}*X\;=\;{\left\{b_{u,n}\right\}}_{n\;=\;0}^{N-1}*{\left\{x_{n}\right\}}_{n\;=\;0}^{N-1}$$ (1)

Where * is element wise multiplication, ***B***_*u*_ are uncorrelated *N*-length phase shift sequence of unity moduli and *u*∈{0,1, …,*U*-1} is the SI index of the phase shift sequences. In the element wise multiplication of two vectors, each element in a given vector is multiplied by its corresponding element in the other vector. For each ***X***_*u*_, its inverse Fourier transform $X_{u}^{'}$ is calculated. The sequence $X_{u}^{'}$ with the lowest PAPR and its corresponding SI index is transmitted.

In the proposed semi-blind SLM, the SI index is embedded in the transmitted symbols using spread spectrum code as follow. *U* sub-sequences moduli of length *M* denoted by ${B_{u}^{'}\;=\;\{b_{u,m}^{'}\}}_{m\;=\;0}^{M-1}$ are generated. Each $B_{u}^{'}\;$ has *k* elements of value *C* to boost the energy of the transmitted sequences and *M*-*k* elements of value *D* = $\sqrt[{2}]{\frac{M-kC^{2}}{M-k}}$ to damp the energy of the transmitted sequences. *C*∈[1,1.5] is known as extension factor and D is known as damping factor. The values of *U*, *M* and *k* are determined based on the utilized spread spectrum code. The generation of $B_{u}^{'}$ is done as follow. For a given PN sequence [–1–1–1 1–1 1 1], the extension factor *C* is used at the 1’s locations and the damping factor *D* is utilized at the -1’s locations. The resulting $B_{u}^{'}$ is [*D D D C D C C*]. Embedding PN sequences in $B_{u}^{'}$ enable the use of the error correction capability of the spread spectrum code in the detection of the SI-index. Bringing in the extension factor *C* and the damping factor *D* changes the average energy per transmitted symbol. The change in the energy for symbols multiplied by *C* is (*C* ^2^−1)*E*[|*x*_*n*_|^2^] while the change in the energy for symbols multiplied by *D* is (*D* ^2^−1)*E*[|*x*_*n*_|^2^], where E[.] designates the expectation operator. Hence, the change in the average energy per transmitted symbol *G*_*new*_, expressed in decibel (dB), is given by

$$G_{new}\;=\;10{log}_{10}\left[\frac{ME[{\left|x_{n}\right|}^{2}]+k\left(C^{2}-1\right)E[{\left|x_{n}\right|}^{2}]+\left(M-k\right)(D^{2}-1)E[{\left|x_{n}\right|}^{2}]}{ME[{\left|x_{n}\right|}^{2}]}\right]$$

$$G_{new}\;=\;10{log}_{10}\left[1+\delta_{C}\left(C^{2}-1\right)+\delta_{D}\left(D^{2}-1\right)\right]$$ (2)

Where *δ*_*C*_ = *k*/*M* and *δ*_*D*_ = (*M*-*k*)/*M*.

In [7,8] and [9], codes which are based on permutations are embedded in the transmitted data using only the extension factor C which changes the average energy per transmitted symbol by a value *G*_*old*_:

$$G_{old}\;=\;10{log}_{10}\left[1+\frac{k}{M}\left(C^{2}-1\right)\right]$$ (3)

**Fig 1:**
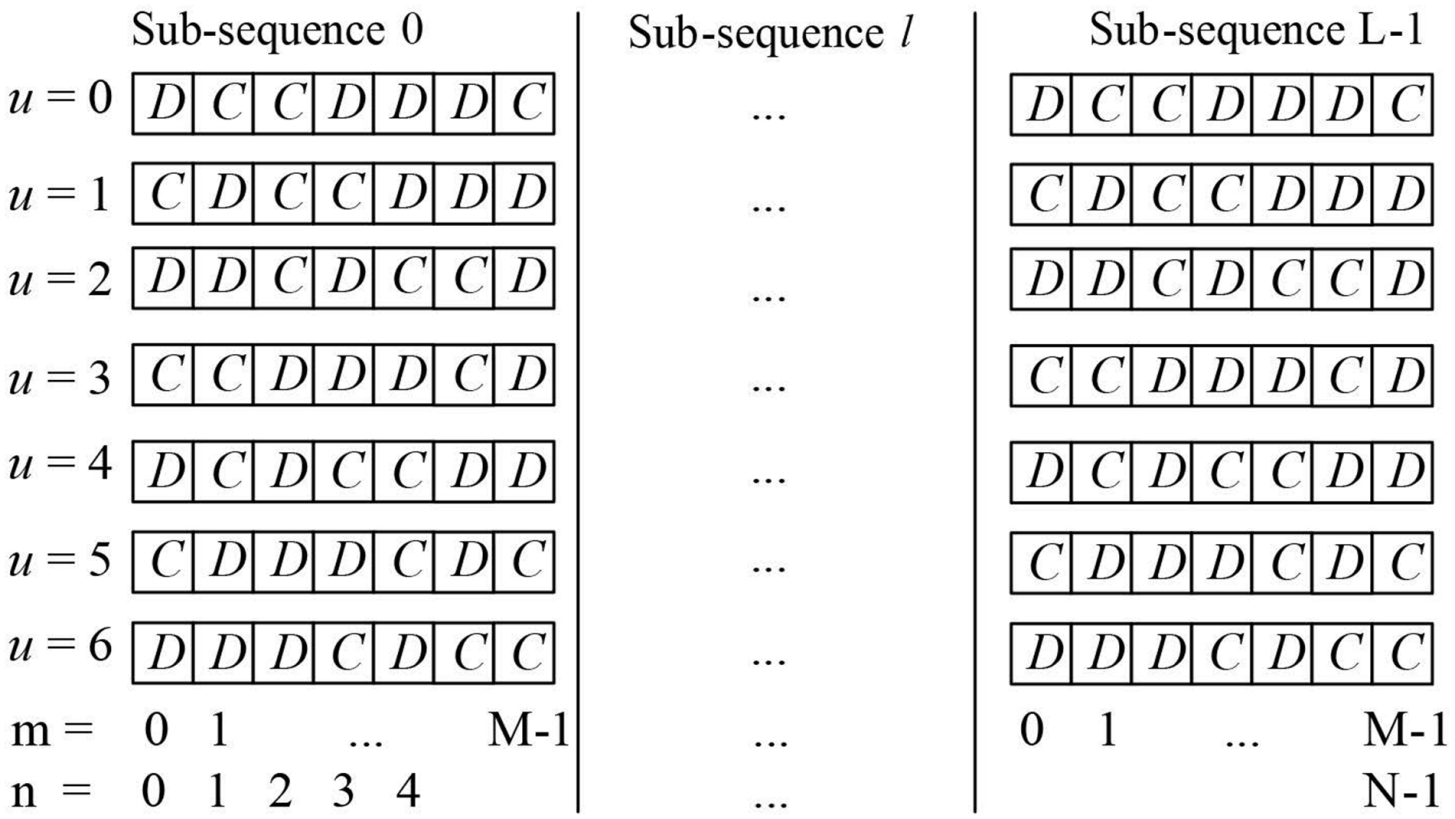
Moduli of Sequences *B*_*u*_ Used in The SLM Technique Proposed.

Unlike [9], the construction of the vectors ***B***_*u*_ is done by generating the phase shift vectors and the moduli vectors separately as in [7]. Each $B_{u}^{'}$ is repeated L = *N*/*M* times to construct the moduli of N length vectors ***B***_*u*_ as illustrated in Fig 1. The phase shifts of the *U* N-length ***B***_*u*_ vectors are chosen to be uncorrelated and uniformly random distributed from 0 to 2*π*. Once ***B***_*u*_ is contracted the rest of the transmitter work exactly as classical SLM. In the proposed receiver, it is assumed that *N* complex symbols which form OFDM data block $X\;=\;{\left\{x_{n}\right\}}_{n\;=\;0}^{N-1}$ have an equally likely randomly distributed energy. The idea behind the proposed receiver is based on the construction of ***B***_*u*_ which results in disparity between energy locations in the received vector.

Algorithm I shows the steps for recovering the SI index in the proposed receiver. In step (1), the received OFDM vector is reshaped to form a matrix *V* of dimension *L*×*M*. The reshaping process guarantees that, *k* columns of the matrix *V* will have the received data elements *y*_*n*_ = *h*_*n*_·*x*_*u*,*n*_+*n*_*n*_ that contain transmitted symbols *x*_*u*,*n*_ having phase shifts with moduli of *C* and *M*-*k* columns will have elements *y*_*n*_ that contain *x*_*u*,*n*_ having phase shifts of moduli *D*.

$$h_{n}\;=\;\sum_{z\;=\;0}^{Z-1}{\overset{`}{h}}_{z}.{exp}^{\frac{-j2\pi nz}{N}}$$ (4)

where ${\overset{`}{h}}_{z}$ is a complex Gaussian sample with zero mean and unit variance representing the fading experienced by the *z* ^th^ tap.

Algorithm I: recovering the SI index in the proposed receiver. Given the OFDM received vector $Y\;=\;{\left\{y_{n}\right\}}_{n\;=\;0}^{N-1}$ and the *U* possible phase shift ***B***_*u*_, the SI index can be recovered as follow

1. Reshaping the received ***Y*** vector to form the matrix ***V***
  $$V=\left[\begin{array}{cccc} v_{0,0} & v_{0,1} & \ldots & v_{0,M-1}\\ v_{1,0} & v_{1,1} & \ldots & v_{1,M-1}\\ \vdots & \vdots & \ldots & \vdots \\ v_{L-1,0} & v_{L-1,1} & \ldots & v_{L-1,M-1} \end{array}\right]\text{ , }v_{l,m}=y_{Ml+m}$$ (5)
  where *l*, *m* are the row and the column index of the matrix V respectively
2. Calculate the average energy per column ***W***
  $$W=\left[\begin{array}{cccc} w_{0} & w_{1} & \ldots & w_{M-1} \end{array}\right]\text{ , }w_{m}=\frac{1}{L}\sum_{l=1}^{L-1}{}_{}{\left|v_{l,m}\right|}^{2}$$ (6)
3. Estimate the moduli of the phase shift sub-sequence
  $$\left|B_{\tilde{u}}^{'}\right|\;=\;\left[\left|b_{\tilde{u,}0}^{'}\right|\;\left|b_{\tilde{u,}1}^{'}\right|\;\ldots \;\left|b_{\tilde{u,}M-1}^{'}\right|\right]$$
  such that
  $$\left|b_{\tilde{u},m}\right|\;=\;\left\{\begin{array}{c} C\\ D \end{array}\;\begin{array}{c} \text{iff}\;w_{m}\in F\\ \text{otherwise} \end{array}\right.$$ (7)
  Where *F* is a set of the *k* maximum values in the vector ***W***
4. Estimate the code ${PN}_{\tilde{u}}$ using $\left|B_{\tilde{u}}^{'}\right|$ by replacing *C* with 1 and *D* with -1.
5. Compared ${PN}_{\tilde{u}}$ with all possible ***PN***_***u***_ using cross-correlation to obtain *α*_*u*_
  $$\alpha_{u}=R_{u}\left[0\right]=\sum_{m\;=\;0}^{M-1}PN_{\tilde{u}}\left[m\right]PN_{u}\left[m\right]$$ (8)
6. The SI index u that gives the maximal value of *α*_*u*_ is considered as received index $\tilde{u}$.
  $$\tilde{u}\;=\;arg\;{\text{ max}}_{u\in \left\{1,2,..,\;U\right\}}\alpha_{u}$$ (9)

The average energy of each column in the matrix *V* is then calculated to introduce a new vector ***W*** as shown in step 2. The locations of the *k* maximum values and the *M-k* minimum values, in the vector ***W***, are corresponding to the locations of the extension factor *C* and the damping factor *D* respectively in the received $B_{\tilde{u}}^{'}$. Once $B_{\tilde{u}}^{'}$ is estimated, ${PN}_{\tilde{u}}$ can be detected as shown in step 3 and 4 respectively. In step 5, the estimated ${PN}_{\tilde{u}}\;$ is cross-correlated with the *U* possible PN sequences. Finally, select $B_{u}^{'}$ which corresponds to ${PN}_{u}^{'}$ that gives maximum *α*_*u*_. For most of the semi-blind SLM systems [4,7,8] and [9], in order to detect the SI index, U comparisons need to be done for each N subcarriers. For example, if number of subcarriers is 1024 (as in commercial DVB systems) and U = 7, the proposed system performs seven comparisons for each 1024 subcarriers. The proposed system has less computational burden than the systems proposed in [4,7,8] and [9]. Furthermore, it does not transmit and demodulate the side information as in the classical SLM. The time required for transmitting and demodulating the SI index in classical SLM or for doing more computations as in other SLM systems can be used for these comparisons. Hence the proposed technique can support the real time communication.

## Analysis of SI Detection Error Rate for the Proposed SLM

The *α*_*u*_ for 1≤*u*≤*U* and $u\neq \;\tilde{u}$ is i.i.d., since *PN*_*u*_[*m*] in (8) is i.i.d. with different *u*. The SIER can be written as in [4]

$$\begin{array}{l} SIER\;=\text{Pr}\left(\underset{u\in \left\{1,2,\ldots ,U\right\},u\neq \;\tilde{u}}{\text{max}}\alpha_{u}>\alpha_{\tilde{u}}\right)\\ \;=1-\text{Pr}\left(\underset{u\in \left\{1,2,\ldots ,U\right\},u\neq \;\tilde{u}}{\text{max}}\left(\alpha_{u}-\alpha_{\tilde{u}}\right)<0\right)\\ \;=1-\prod_{1\;\leq \;u\;\leq \;U,\;\;\;u\neq \;\tilde{u}}F_{\alpha_{u}-\alpha_{\tilde{u}}}\left(0\right)\\ \;=1-{\left(F_{\alpha_{u}-\alpha_{\tilde{u}}}\left(0\right)\right)}^{U-1} \end{array}$$ (10)

where *F*(·) is a cumulative distribution function and $\left(\alpha_{u}-\alpha_{\tilde{u}}\right)$ for 1≤*u*≤*U* and $u\neq \;\tilde{u}$ with common $\alpha_{\tilde{u}}$ is an i.i.d. random variable. For $\left(\alpha_{u}-\alpha_{\tilde{u}}\right)$ to be less than zero, means that a correct decision in (8) occurs. Recall (6)

$$\begin{array}{l} w_{m}=\frac{1}{L}\overset{L}{\underset{l=1}{\sum }}{\left|v_{l,m}\right|}^{2}=\frac{1}{L}\overset{L}{\underset{l=1}{\sum }}{\left|y_{Ml+m}\right|}^{2}\\ \;\;=\frac{1}{L}\sum_{l=1}^{L}{\left|h_{Ml+m}\cdot x_{\tilde{u},Ml+m}+n_{Ml+m}\right|}^{2} \end{array}$$ (11)

Let

$$w_{m}^{\left(C\right)}\;=\;\frac{1}{L}\sum_{l\;=\;1}^{L}{\left|h_{Ml+m}∙{Cx}_{Ml+m}e^{j\varphi_{Ml+m}}+n_{Ml+m}\right|}^{2},$$

$$w_{m}^{\left(D\right)}\;=\;\frac{1}{L}\sum_{l\;=\;1}^{L}{\left|h_{Ml+m}\cdot Dx_{Ml+m}e^{j\varphi_{Ml+m}}+n_{Ml+m}\right|}^{2}$$ (12)

Thus

$$F_{\alpha_{u}-\alpha_{\tilde{u}}}\left(0\right)\;=\;\text{Pr}\left(w_{m_{1}}^{\left(D\right)}<w_{m_{2}}^{\left(C\right)}\right)\;=\;\text{Pr}\left(w_{m_{1}}^{\left(D\right)}-w_{m_{2}}^{\left(C\right)}<0\right),$$ (13)

with *n*_*n*_ a complex zero-mean Gaussian noise and *h*_*n*_ as in (4), then the probability distribution function $f_{w_{m_{1}}^{\left(D\right)}}\left(w\right)\;and{\;f}_{w_{m_{2}}^{\left(C\right)}}\left(w\right)$ has Rayleigh distribution. The SIER can be written as

$$SIER\;=1-\;{\left(\int_{-\infty }^{0}f_{w_{m_{1}}^{\left(D\right)}-w_{m_{2}}^{\left(C\right)}}\left(w\right)dw\right)}^{U-1}$$ (14)

## System analysis and Simulation results

The proposed system is evaluated over fading channel which assumed to be quasi-static frequency-selective Rayleigh fading channel with two different setups for comparison purpose. In the first setup the channel has *Z* = 4 equal-power taps as in [9] while in the second the channel has Z = 6 different power level taps as in [4]. In the simulation, prefect CSI is only assumed in the detection of the SI for the receivers presented in [4,7] and [9]. At the transmitter output, it is assumed the use of a nonlinear power amplifier (PA) simulated using Rapp’s model [11]. The parameters of the nonlinear PA are the smoothness parameter *p* = 3 and the input backoff (IBO) = 7dB. The system parameters considered in the conducted simulations are listed in Table 1.

**Table 1:** System parameters.

Proposed SLM Parameters	N = 70, 126, 252, 511, 1022
	U = 7, M = 7, K = 3
SLM [4] parameters	N = 1024
	U = 8, N_p_ = 64, N_d_ = 960
SLM [7] parameters	N = 1024
	U = 8, M = 8, K = 3
SLM [9] parameters	N = 70,125,255,510
	U = 10, M = 5, K = 2

In [4,7],[9] and in this paper, the system parameters are based on the selected code and hence it is impossible for all systems to have the same parameters. In [7,9] and this paper, the N length moduli vector ***B***_*u*_, which is used to embed the SI index in the N transmitted subcarriers, is generated by repeating the M length sub-sequence moduli $B_{u}^{'}$ for L times. The sub-sequence moduli $B_{u}^{'}$ are generated based on the utilized code. The proposed system uses the PN sequence which has a length 2^n^-1, where n is one of the parameters used to describe the PN sequence and is equal to the length of the shift register used to generate the PN sequence. On the other hand, the codes used by [9] and [7] are based on permutation. Hence, it is impossible for all SLM systems to have the same number of subcarriers N. However, number of subcarriers has been chosen to be very close to the one selected in [7] and [9] as shown in Table 1. The authors in [9], states that the code length M should be minimized to minimize the probability of SI detection error. In [7]and [9], M is equal to 8 and 5 respectively while in this paper M is equal to 7. Moreover, increasing number of utilized code U results in better probability of the PAPR reduction [9]. In [4,7] and [9], U is equal to 8, 8 and 10 respectively while in this paper U is equal to 7. By comparing the code length M and the number of codes U for techniques presented in [4,7], [9] and the proposed technique, it can be seen that these parameters were settled to the benefit of the techniques proposed in [4,7] and [9]. In [5] and [9], it has been shown, that the bit error rate (BER) of the SLM is sensitive to modulation techniques which have unequal energy per symbol. Hence, all the simulation results are obtained using the 16 Quadrature Amplitude modulation (QAM).

### A. Energy consumption

The moduli sub-sequences utilized in this paper are given in Fig 1. Each of these subsequences has *k* = *3* elements of value *C* used for energy boosting and *M*-*k* = 4 elements of value *D* used for energy damping. By solving (2) for the utilized codes, it can be seen that at *C* = 1.286 the total boosted energy is equal to the total damp energy and hence *G*_*new*_ = 0dB. This *C* value is used to obtain results for the proposed SLM. Results for the SLM techniques introduced in [7] and [9] are obtained using *C* = 1.2 as it has been selected by the authors of these papers. It can be seen from (3) that such value of *C* results in increasing the average energy per transmitted symbol by *G*_*old*_ = 0.663 dB and 0.704 dB in the SLM technique proposed in [7] and [9] respectively.

### B. Computational complexity

The receiver presented in [7] and [9] carries out (UN+5N)/4 complex multiplications per frame for the detection of the SI index as it can be seen from (2) in [7]. Eqs (9) and (10) presented in [4] show that the pilot-aided SLM receiver carriers out 2UN_p_-U complex multiplications of the detection of the SI index, where N_p_ = N-N_d_, N_p_ is number of pilot symbols and N_d_ is number of data symbols. On the other hand, the SI index detection for the proposed SLM comprises the following computational complexity:

- Number of real multiplication in (6) is 2N
- Number of real multiplication in (8) is MU

Hence the overall complex multiplication in the detection of SI index for the proposed SLM is (2N+MU)/4 = N/2 + MU/4.. For N = 1024 and U = 8, the receiver presented in [7,9] and [4] carries out 3328 and 1016 complex multiplications respectively while the proposed receiver caries out 526 complex multiplications.

### C. PAPR performance

**Fig 2:**
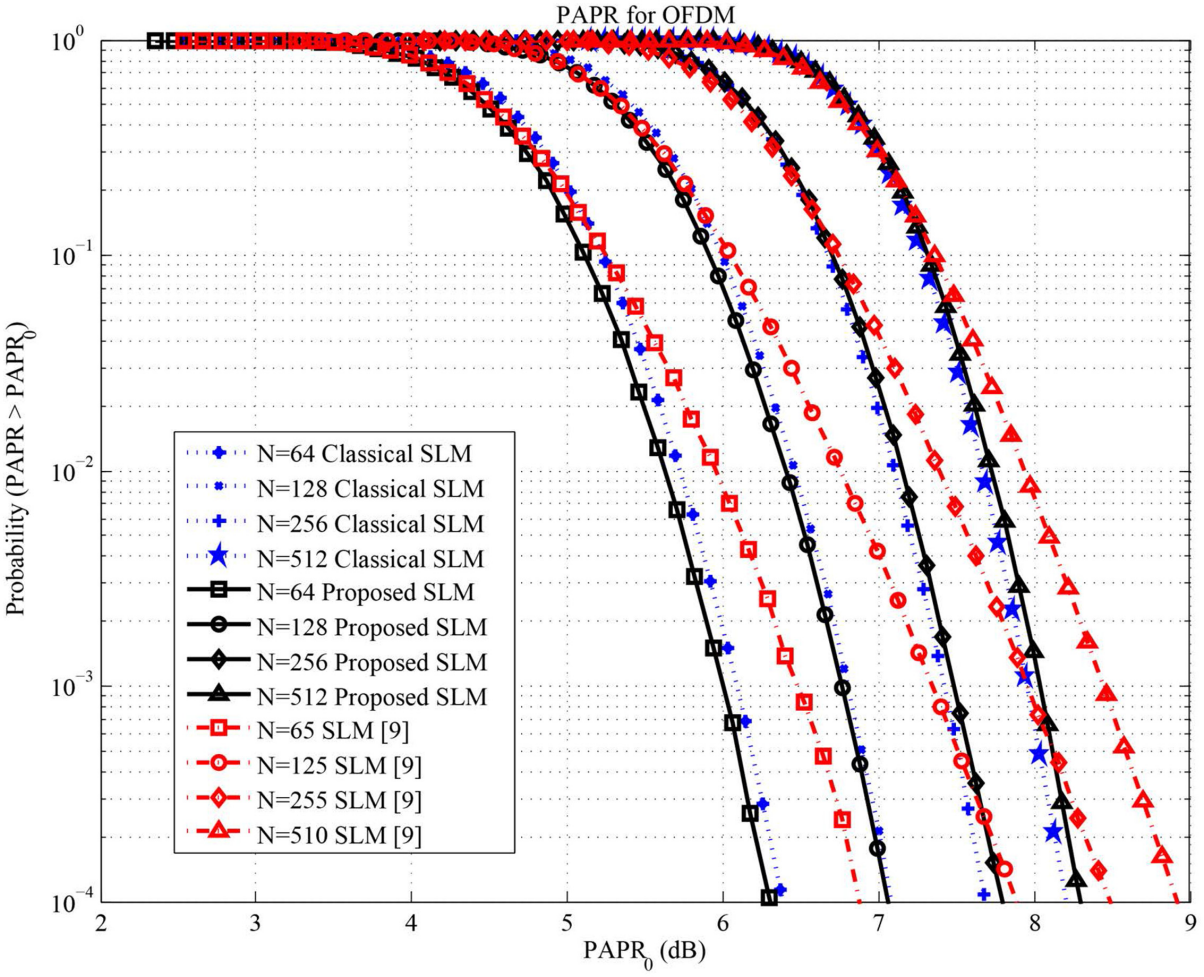
CCDF of the PAPR obtained with the proposed SLM system, classical SLM and the SLM introduced in [9] with oversampling factor of 4 and Z = 4.

Simulation results are obtained using oversampling factor equal to 4. Fig 2 shows the complementary cumulative distribution function (CCDF) of the PAPR achieved with the proposed semi-blind SLM transmitter compared to the SLM transmitter in [9] and classical SLM technique. The proposed semi-blind SLM technique has PAPR lower than the SLM technique in [9]. This is because the phase shift vectors are selected to be aperiodic over all vectors only the moduli are periodic as in [7] while in [9] the phase vectors are periodic. Moreover, the proposed SLM has PAPR reduction similar to the classical SLM.

### D. Probability of SI detection error

**Fig 3:**
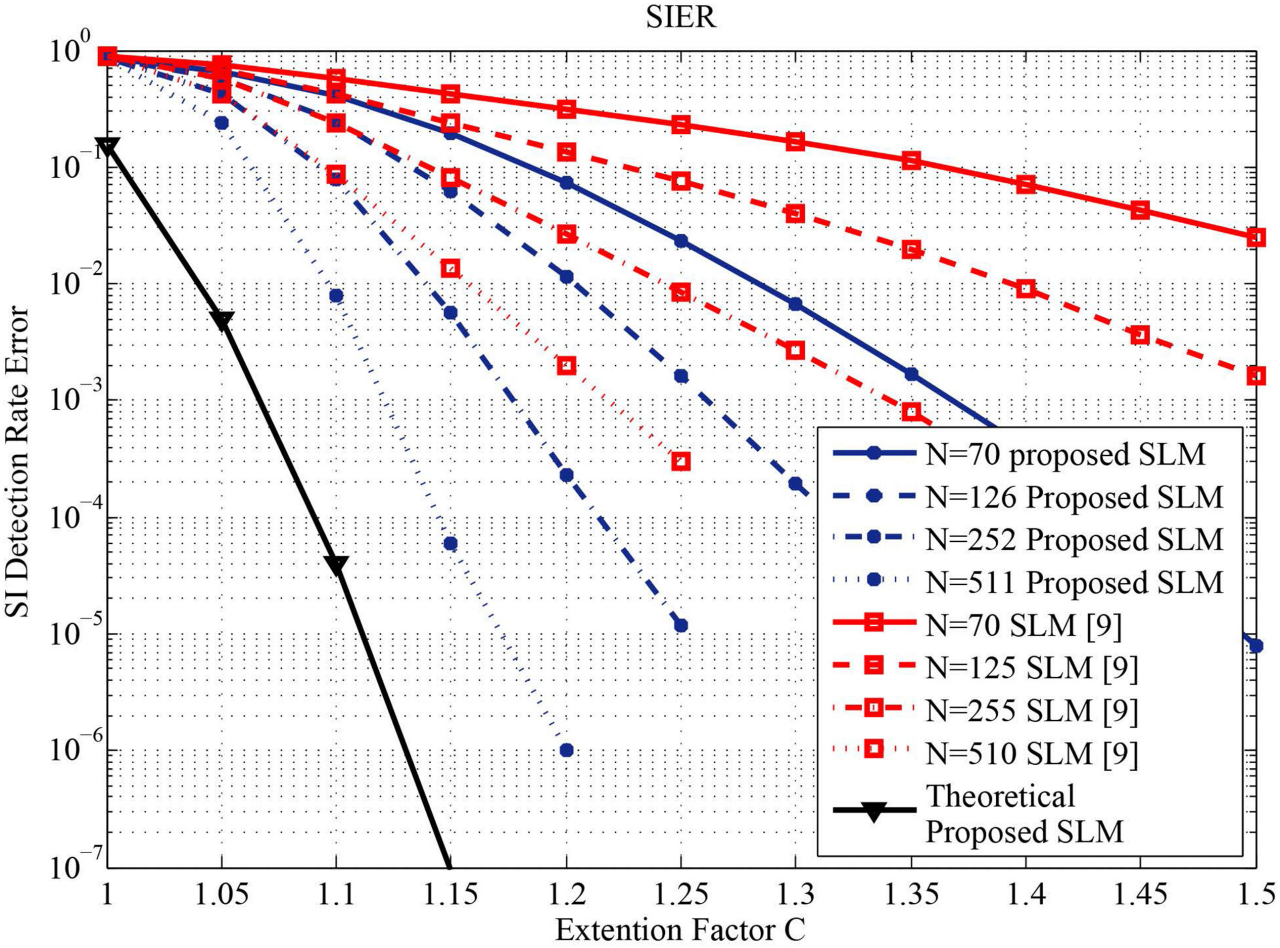
Probability of SI detection error *P*_de_ as a function of the extension factor C for both the proposed SLM and the SLM introduced in [9] using 16 QAM and Z = 4.

All results in this subsection are obtained for symbol energy to noise ratio ***E***_***s***_ **/*N***_***o***_ = 10 dB. The probability of SI detection error ***P***_***de***_ is compared over four different numbers of subcarriers *N* as listed in Table 1. Fig 3 shows the probability of SI detection error ***P***_***de***_ as a function of the extension factor *C* for the proposed system and the system introduced in [9]. The simulation results for the proposed system are obtained for the extension factor C = 1.286. Such an extension factor results in zero increase in average energy per transmitted symbol as it can be from Eq (2). However increasing the extension factor results in better performance for both systems as it can be seen from Fig 3, the average energy per transmitted symbol is increased. Note that, the increment in the energy due to increasing C is smaller in the case of the proposed system than the systems proposed in [7] and [9] as it can be seen from (2) and (3). Moreover, increasing *N* decreases the probability of SI detection error. Fig 3 also illustrates that the proposed system can achieve better ***P***_***de***_ than the one accomplished by the system introduced in [9]. Fig 3 also shows the theoretical SIER of the proposed SLM system based on (14) at a large number of subcarriers N.

### E. Bit error rate performance

**Fig 4:**
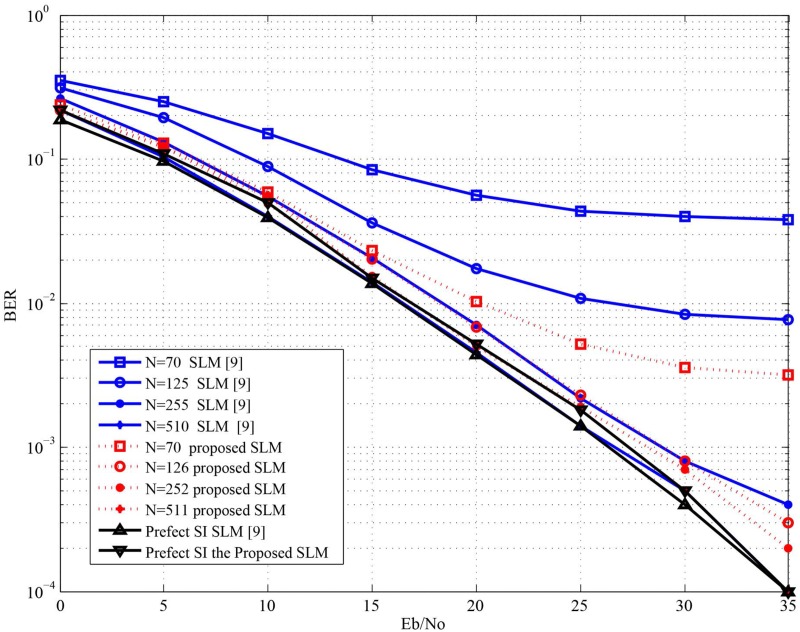
Comparison between BER performance for the proposed SLM system and the SLM system introduced in [9] using 16 QAM and Z = 4.

The effect of the proposed SLM on the BER performance is one of the major aspects that need to be investigated. Fig 4 shows the BER performance versus ***E***_***b***_ **/*N***_***o***_ curves obtained with the proposed receiver compared to the receiver introduced in [9]. It can be seen from Fig 4 that the proposed system outperforms the system introduced in [9] for *N* = 70,125,255. For *N* = 510 the system proposed in [9] is slightly better than the proposed system.

**Fig 5:**
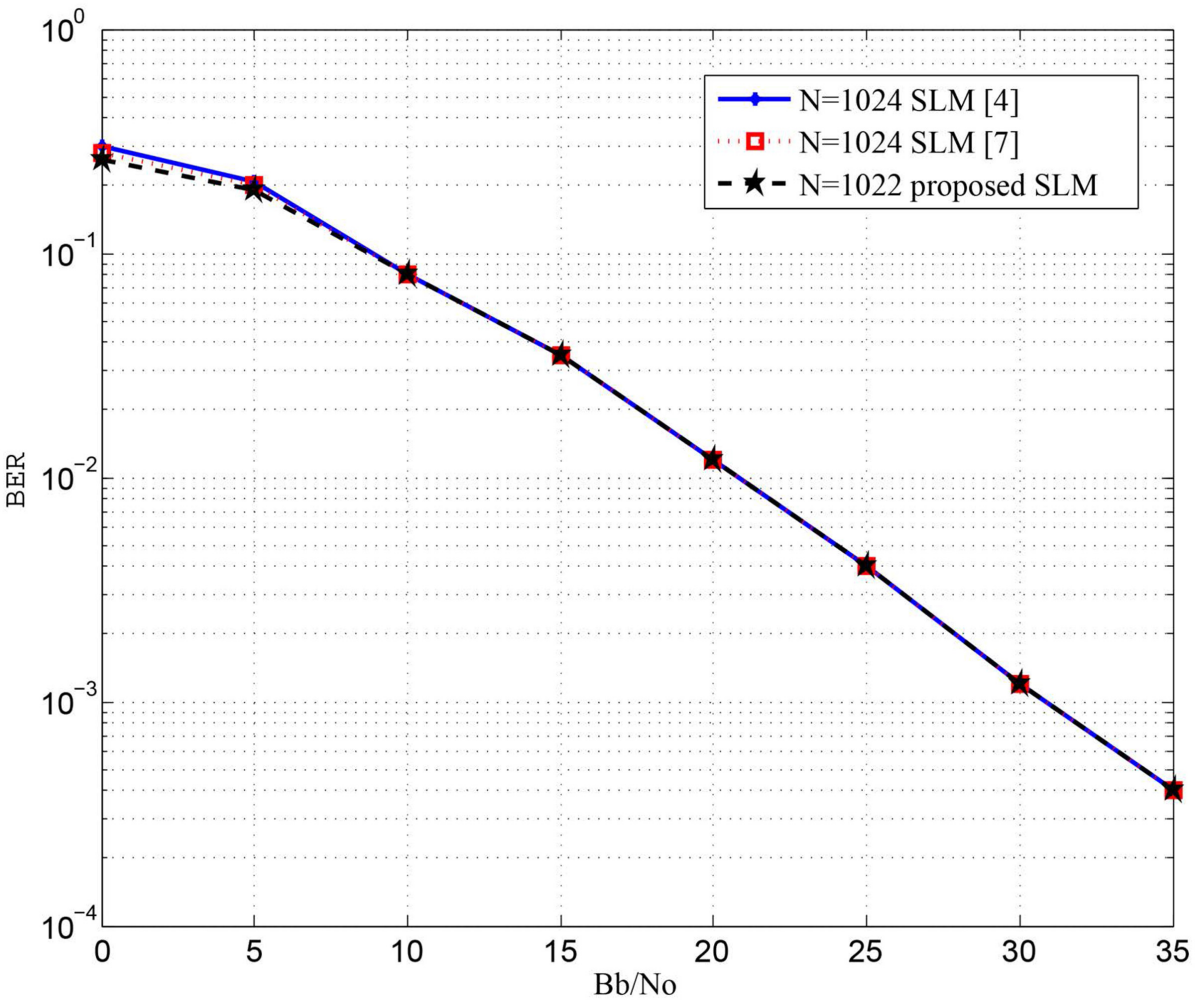
Comparison between BER performance for the proposed SLM system and the SLM system introduced in [4] using 16 QAM and Z = 6.

However, from Fig 3 it can be noted that the probability of SI detection error for *N* = 510 much better in case of the proposed system. This can be justified by comparing both systems at perfect SI detection. It can be seen from Fig 4 that BER at perfect SI index detection for the system proposed in [9] is slightly better that of the proposed system. This small degradation in the BER for the proposed system is due to damping the energy of some of the transmitted symbols. Nevertheless, the proposed system uses no extra energy for embedding the SI index and has less computational complexity. Fig 5 shows the BER performance versus *E*_*b*_/*N*_*o*_ curves obtained with the proposed SLM compared to the SLM introduced in [4] and [7]. It can be show form Fig 5 that the proposed SLM leads to BER performance close to or better than the SLM presented in [4] and [7].

Furthermore, the theoretical superiority of the proposed approach in term of the BER can be demonstrated as follow. If the transmission channel over each $\hat{M}$-QAM subcarrier can be modeled as AWGN, the bit error probability BER obtained with the proposed SLM method in the absence of any SI detection error can then be expressed as

$${BER}_{Perfect\;SI}\;=\;\frac{4k}{{Mlog}_{2}\hat{M}}\left(1-\frac{1}{\sqrt{\hat{M}}}\right)Q\left(\sqrt{\frac{3C^{2}{log}_{2}\hat{M}\;\frac{E_{b}}{N_{o}}}{\hat{M}-1}}\right)+\frac{4\left(M-k\right)}{{Mlog}_{2}\hat{M}}\left(1-\frac{1}{\sqrt{\hat{M}}}\right)Q\left(\sqrt{\frac{3{D^{2}log}_{2}\hat{M}\;E_{b}/N_{o}}{\hat{M}-1}}\right)$$ (15)

If the SI index gets corrupted, then the recovered OFDM symbols may be completely erroneous. In this case, the recovered symbols can be considered as the output of a noisy channel where half of bits is corrupted and hence *BER*_*Corrupted SI*_ = 0.5. The overall BER can be expressed as

$$BER\;=\;\left(1-SIER\left(C,D,E_{b}/N_{o}\right)\right){BER}_{Perfect\;SI}+\;SIER\left(C,D,E_{b}/N_{o}\right){BER}_{Corrupted\;SI}$$ (16)

$$BER\;=\;\left(1-SIER\left(C,D,E_{b}/N_{o}\right)\right)\left[\frac{4k}{{Mlog}_{2}\hat{M}}\left(1-\frac{1}{\sqrt{\hat{M}}}\right)Q\left(\sqrt{\frac{3C^{2}{log}_{2}\hat{M}\;\frac{E_{b}}{N_{o}}}{\hat{M}-1}}\right)+\frac{4\left(M-k\right)}{{Mlog}_{2}\hat{M}}\left(1-\frac{1}{\sqrt{\hat{M}}}\right)Q\left(\sqrt{\frac{3{D^{2}log}_{2}\hat{M}\;E_{b}/N_{o}}{\hat{M}-1}}\right)\right]+0.5\;SIER\left(C,D,E_{b}/N_{o}\right)$$ (17)

Similarly for the system proposed in [9]

$$BER\;=\;\left(1-SIER\left(C,E_{b}/N_{o}\right)\right)\left[\frac{4k}{{Mlog}_{2}\hat{M}}\left(1-\frac{1}{\sqrt{\hat{M}}}\right)Q\left(\sqrt{\frac{3C^{2}{log}_{2}\hat{M}\;E_{b}/N_{o}}{\left(\hat{M}-1\right)}}\right)+\frac{4\left(M-k\right)}{{Mlog}_{2}\hat{M}}\left(1-\frac{1}{\sqrt{\hat{M}}}\right)Q\left(\sqrt{\frac{3{log}_{2}\hat{M}\;E_{b}/N_{o}}{\left(\hat{M}-1\right)}}\right)\right]+0.5\;SIER\left(C,E_{b}/N_{o}\right)$$ (18)

At $\hat{M}$ = 16 and *E*_*s*_/*N*_*o*_ = 10dB which is equivalent to *E*_*b*_/*N*_*o*_ = 3.98dB, the proposed SLM system BER is equal to 0.0823 for number of subcarrier N = 70 with SIER = 0.01 at C = 1.286. On the other hand, the system in [9] has BER equal to 0.18415 for number of subcarrier N = 70 with SIER = 0.3 at C = 1.2. Hence, the BER obtained by the SLM introduced in [9] is higher than the BER obtained by the SLM proposed in this paper.

## Conclusion

In this paper an innovative OFDM selected mapping system is proposed. At the transmitter, spread spectrum codes are embedded in the transmitted symbol with a novel manner which does not increasing the average energy per transmitted symbol. The detection of the SI index is based on maximizing the disparity between energy locations in the received vector. It has the advantages that CSI is not required for the SI detection, low computational complexity and it outperforms the SLM receivers introduced in [4,9]. Simulation results show that the proposed receiver can achieve better probability of SI detection error P_de_ than that obtained by the system introduced in [9]. Furthermore, the proposed system gives better BER or equal than the systems introduced in [4] and [9].
